# Gli2 and Gli3 Localize to Cilia and Require the Intraflagellar Transport Protein Polaris for Processing and Function

**DOI:** 10.1371/journal.pgen.0010053

**Published:** 2005-10-28

**Authors:** Courtney J Haycraft, Boglarka Banizs, Yesim Aydin-Son, Qihong Zhang, Edward J Michaud, Bradley K Yoder

**Affiliations:** 1 Department of Cell Biology, University of Alabama, Birmingham, Alabama, United States of America; 2 University of Tennessee Oak Ridge National Laboratory Graduate School of Genome Science and Technology, University of Tennessee, Knoxville, Tennessee, United States of America; 3 Life Sciences Division, Oak Ridge National Laboratory, Oak Ridge, Tennessee, United States of America; Stanford University School of Medicine, United States of America

## Abstract

Intraflagellar transport (IFT) proteins are essential for cilia assembly and have recently been associated with a number of developmental processes, such as left–right axis specification and limb and neural tube patterning. Genetic studies indicate that IFT proteins are required for Sonic hedgehog (Shh) signaling downstream of the Smoothened and Patched membrane proteins but upstream of the Glioma (Gli) transcription factors. However, the role that IFT proteins play in transduction of Shh signaling and the importance of cilia in this process remain unknown. Here we provide insights into the mechanism by which defects in an IFT protein, Tg737/Polaris, affect Shh signaling in the murine limb bud. Our data show that loss of *Tg737* results in altered Gli3 processing that abrogates Gli3-mediated repression of Gli1 transcriptional activity. In contrast to the conclusions drawn from genetic analysis, the activity of Gli1 and truncated forms of Gli3 (Gli3R) are unaffected in *Tg737* mutants at the molecular level, indicating that Tg737/Polaris is differentially involved in specific activities of the Gli proteins. Most important, a negative regulator of Shh signaling, Suppressor of fused, and the three full-length Gli transcription factors localize to the distal tip of cilia in addition to the nucleus. Thus, our data support a model where cilia have a direct role in Gli processing and Shh signal transduction.

## Synopsis

Cilia are small projections extending from the surface of most cells. Research has shown that they are important in diseases such as cystic kidney diseases as well as during the development of many tissues including the limb. More recently, proteins such as Polaris, which is required to build cilia, have been demonstrated to be essential for the regulation of Sonic hedgehog (Shh) signaling, although the mechanism has remained elusive. Precise regulation of Shh signal transduction is important for the proper development of many tissues. Excessive activation of the Shh pathway results in severe developmental defects and has been implicated in certain types of cancer. In the limb, Shh signaling is involved in digit development, and excess signaling leads to the formation of extra digits. The main targets of Shh signaling are the Glioma (Gli) family of transcription factors, and Gli3 has been shown to be processed to a shortened repressor form when Shh signaling is repressed. The localization of the Gli transcription factors and Suppressor of fused, a protein involved in the regulation of Gli protein function, to cilia suggests that the cilia may be an important site for regulation of Shh signal transduction by modulating Gli protein function.

## Introduction

Cilia are microtubule-based organelles that protrude from the surface of most cells in the mammalian body and are formed through a conserved process termed intraflagellar transport (IFT) [1]. Polaris, the protein encoded by *Tg737,* is a core component of the mammalian IFT machinery and is required for the formation of all cilia and flagella [2,3]. Mice homozygous for the hypomorphic *Tg737^orpk^* allele exhibit phenotypes in many tissues including the formation of cysts in the kidney, liver, and pancreas, hydrocephalus, and skeletal patterning defects that include extra molar teeth, cleft palate, and preaxial polydactyly [2,4–6]. While *Tg737^orpk^* mutants are viable, complete loss of *Tg737* function in *Tg737* ^Δ2–3β-gal^ mutants results in midgestation lethality, randomization of the left–right body axis, neural tube closure and patterning defects, and formation of eight to ten unpatterned digits per limb [3]. In *Tg737^orpk^* and *Tg737* ^Δ2–3β-gal^ mutant mice, cilia are severely malformed or absent, respectively, suggesting that this organelle is required for normal development and patterning of many tissues in the mammalian body [2,3,5].

The mammalian limb is patterned through the interaction of three main signaling centers [7]. The apical ectodermal ridge is necessary for proper limb outgrowth and proximal–distal length while the surface ectoderm regulates dorsal–ventral patterning. The zone of polarizing activity, located in the posterior mesenchyme, is involved in anterior–posterior patterning including the formation of five digits per limb. Sonic hedgehog (Shh) is secreted by cells in the zone of polarizing activity, and many polydactyl mutants in the mouse have been shown to have ectopic expression of either Shh or genes activated by Shh. The main targets of Shh signaling are the Glioma (Gli) transcription factors [8]. Three Gli transcription factors (Gli1, Gli2, and Gli3) have been identified in mammals. Gli3 exists as a full-length “activator” (Gli3A) that is proteolytically processed into a smaller form with repressor activity (Gli3R) in the absence of Shh ligand [9]. Binding of Shh to its receptor Patched1 (Ptch1) leads to the derepression of Smoothened and blocks processing of the Gli3 transcription factor.

While mutations in Gli1 or Gli2 alone have no affect on digit patterning, loss of one or both alleles of Gli3 in *Gli3^Xt-J^* mice is sufficient to produce ectopic digits [10,11]. The severe polydactyly in *Gli3^Xt-J^* homozygous mutants is associated with ectopic expression of Shh and its target genes [12]; however, the loss of Shh in *Shh^−/−^;Gli3^Xt-J^* double mutants results in identical digit patterning defects as seen in *Gli3^Xt-J^* mutants alone, leading to the hypothesis that a pentadactyl restraint is imposed on the limb by Shh counteraction of Gli3 repressor activity [13,14].

Another important component of the Shh signaling pathway involved in Gli protein regulation is Suppressor of fused (Sufu). Sufu is a negative regulator of Shh signaling that interacts with all three Gli proteins and mediates their nuclear export in the absence of Shh [15–17]. In *Drosophila,* Su(fu) is thought to link Ci (Gli homolog) to the ubiquitin proteasome required for conversion of Ci to the small repressor form, as well as retain unprocessed Ci in the cytoplasm in the absence of ligand; however, this has not yet been demonstrated for the mammalian pathway [8].

Previously, we demonstrated that partial disruption of IFT function in *Tg737^orpk^* mutants results duplication of digit I, while complete loss of IFT in *Tg737* ^Δ2–3β-gal^ mutants leads to the formation of up to ten unpatterned digits per limb [6]. Despite the formation of excess digits and a known involvement of Shh in ectopic digit formation in many mouse models [7], *Shh* expression is not altered in either *Tg737^orpk^* or *Tg737* ^Δ2–3β-gal^ mutants [6]. In addition, no alterations in *Ptch1* expression in *Tg737^orpk^* hypomorphic mutants is observed. Recent evidence from the labs of Anderson and Niswander has shown that IFT proteins, including *Tg737/*Polaris, are essential for Shh signaling in both neural tube and limb patterning at the level of Gli3 processing, although the connection between IFT and this signaling pathway remains enigmatic [18–20]. Previous models have speculated that there is a cytosolic role for the IFT proteins or that the cilia generate a signal affecting the activity of the Gli transcription factors. However, in this report we provide evidence supporting the hypothesis that there is a direct role for cilia in Shh signal transduction. Despite normal *Shh* expression in homozygous *Tg737* ^Δ2–3β-gal^ mutant limbs [6], expression of the Shh downstream targets such as *Ptch1* and *Gli1* is lost. Furthermore, cells isolated from mutant limb buds are unable to respond to ShhN conditioned medium (ShhN-CM) in vitro. Even though mutant cells are unable to respond to ShhN-CM, the pathway can be activated by exogenously expressed Gli1; however, Gli2 and full-length Gli3 were found to be inactive in the absence of Polaris. Although full-length Gli3 was nonfunctional in *Tg737* ^Δ2–3β-gal^ mutant cells, expression of a processed form of Gli3 (Gli3R) acts as a potent repressor of Gli1-mediated induction of *Ptch1* expression. Additionally, the amount of full-length Gli3 was markedly increased in *Tg737* ^Δ2–3β-gal^ mutant embryos relative to wild-type controls, suggesting that loss of Polaris results in inefficient Gli3 processing. Most importantly, the data indicate that all three full-length Gli proteins along with Sufu colocalize to the distal tips of cilia in primary limb bud cells. Together our data support a direct role for cilia in the mammalian hedgehog signaling pathway and raise the intriguing possibility that the tip of the cilium is a specialized domain in which proteolytic machinery is concentrated for processing and regulating the activity of Shh signal transduction.

## Results

### Cilia Are Present on Both Ectoderm and Mesenchyme Cells of the Limb Bud

**Figure 1:**
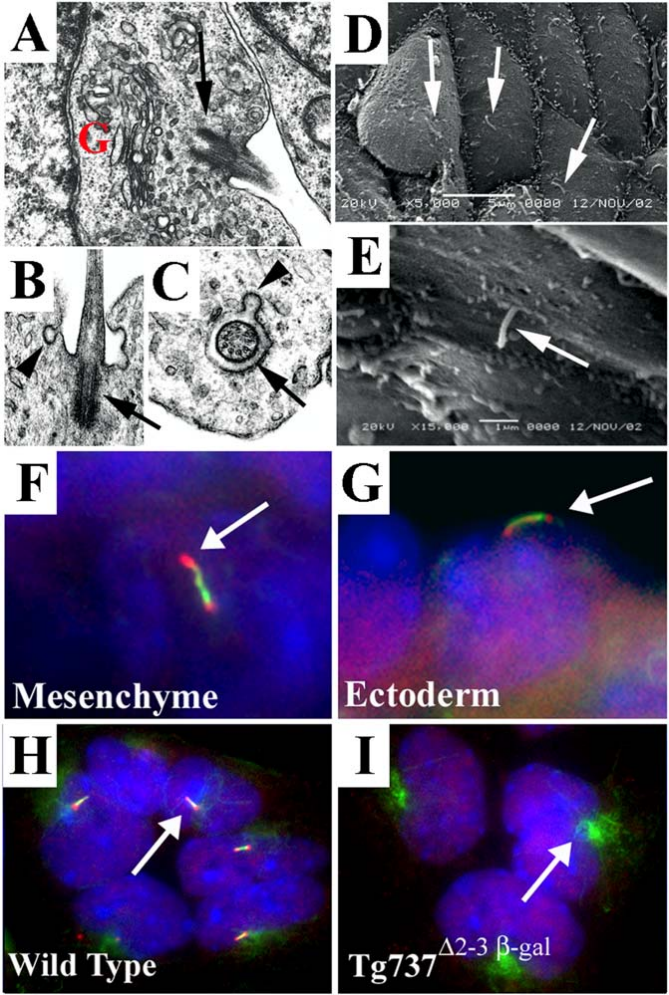
Cilia Are Present on Both Mesenchymal and Ectodermal Cells of the Developing Limb (A–C) Transmission electron micrographs of limb bud mesenchyme show cilia (arrows) closely associated with the Golgi apparatus (“G”). The cilia exhibited a 9 + 0 structure (C) and are often found in deep depressions in the membranes (B). Frequently, small vesicles are observed fusing or budding with the surrounding membrane (arrowheads in [B] and [C]). (D and E) Scanning electron micrographs of the limb ectoderm show a single cilium (arrows) on nearly all ectodermal cells. (F and G) Immunolocalization of Polaris (red) and acetylated α-tubulin (green) in frozen sections of limb buds shows that Polaris concentrates at the base and tip of the axoneme in both mesenchymal (F) and ectodermal (G) cells. Nuclei are blue. (H and I) In primary cultures of cells isolated from E11.5 limb buds, cilia (arrow in H) are also present when visualized with anti-acetylated α-tubulin (green) and anti-Polaris (red) antisera (H). Cilia are absent on cells isolated from *Tg737* ^Δ2–3β-gal^ mutant limb buds (I); however, the stabilized microtubules were still evident around the basal body region (arrow). The nuclear staining for Polaris is present in the *Tg737* ^Δ2–3β-gal^ cells, indicating that it is nonspecific. Nuclei are blue.

To determine whether cilia are present on the developing mouse limb bud, we conducted electron microscopic analysis of embryonic day 11.5 (E11.5) limb buds. Using transmission electron microscopy, cilia were found on the mesenchyme. These cilia have a 9 + 0 microtubule structure, were frequently found in depressions in the cell membrane, and were always closely associated with the Golgi apparatus (Figure 1A–1C). In addition, small vesicular structures were frequently detected near the base of the cilium. Using scanning electron microscopy, we also determined that most, if not all, ectodermal cells exhibit a single cilium (Figure 1D and 1E).

To further confirm the presence of cilia in the limb bud, we conducted immunofluorescence analysis of frozen sections using anti-acetylated α-tubulin, which recognizes stabilized microtubules including the cilium axoneme, and anti-Polaris antiserum. The data indicate that Polaris concentrates at the base and distal tip of cilia on both ectodermal and mesenchymal cells as well as in a punctuate pattern overlapping that of acetylated α-tubulin in the axoneme (Figure 1F–1H). In primary cultures of limb bud cells, cilia were found on most cells when visualized with anti-acetylated α-tubulin and anti-Polaris antibodies (Figure 1H). In contrast, the cilia were completely absent from cells isolated from *Tg737* ^Δ2–3β-gal^ mutants (Figure 1I). Domains of stabilized microtubules were still present around the microtubule organizing center (MTOC) from which the cilia would have emerged.

### The Hedgehog Signaling Pathway Is Repressed in *Tg737* ^Δ2–3β-gal^ Mutants

**Figure 2:**
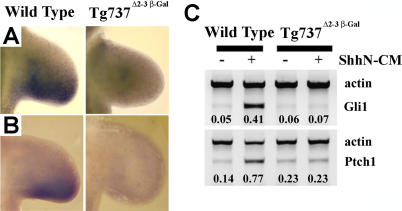
Shh Signaling Is Defective in *Tg737* ^Δ2–3β-gal^ Mutants (A and B) In situ hybridization analysis of *Ptch1* (A) and *Gli1* (B) expression indicates that they are not expressed in the posterior limb buds of *Tg737* ^Δ2–3β-gal^ mutant embryos (E10.5; right panels) as they are in wild-type controls (E10.5, left panels). (C) Incubation of wild-type limb bud cells with ShhN-CM results in upregulation of *Gli1* and *Ptch1* expression (left lanes) compared to vector conditioned medium, whereas no increase is seen in cells isolated from *Tg737* ^Δ2–3β-gal^ mutant limb buds (right lanes). The relative levels of induction standardized to actin are indicated below each lane.

In agreement with previous data in the limb and neural tube [18–20], there was no significant expression of two downstream targets of Shh signaling, *Ptch1* and *Gli1,* in *Tg737* ^Δ2–3β-gal^ null mutant limb buds (Figure 2A and 2B). These data suggest that despite normal Shh expression in these mutants, Shh release or reception is impaired because of the loss of Polaris. These results confirm our assessment that the IFT mutant limb phenotype is not due to ectopic activation of the Shh pathway and that the phenotype in *Tg737* ^Δ2–3β-gal^ mutants resembles that of *Gli3 ^−/−^;Shh^−/−^* embryos [13,14].

### Cells Lacking Polaris Are Unable to Respond to ShhN

To test whether Polaris is required for Shh reception, we isolated cells from *Tg737* ^Δ2–3β-gal^ mutant and wild-type limb buds (E11.5) and cultured the cells in ShhN-CM. The ability of the cells to respond to ShhN was determined by induction of *Ptch1* and *Gli1* expression using semi-quantitative reverse transcription PCR (RT-PCR). While robust response to ShhN-CM was seen in wild-type cells, cells lacking *Tg737* showed no increase in the expression of *Ptch1* or *Gli1* relative to control treated cells (Figure 2C). These data indicate that Polaris is required in Shh responding cells to activate the Shh signaling pathway in the presence of ligand.

### Loss of Polaris Results in Altered Gli Activity and Processing

**Figure 3:**
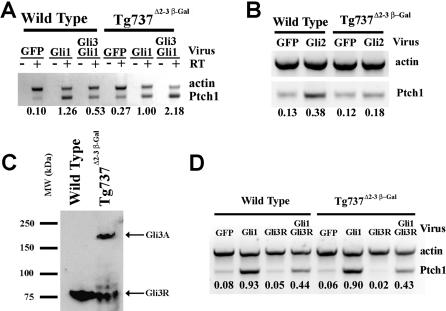
Gli2 and Full-Length Gli3 Function Is Disrupted in *Tg737* ^Δ2–3β-gal^ Mutant Cells (A) Infection of primary limb bud cells (E11.5) with Gli1::GFP expressing adenovirus induces increased *Ptch1* transcription in wild-type cells when compared to infection with GFP-only virus (GFP). Coinfection of wild-type cells with Gli1::GFP and Gli3::GFP results in a decrease in the level of *Ptch1* expression when compared to cells infected with Gli1::GFP only. As seen in wild-type cells, infection of *Tg737* ^Δ2–3β-gal^ mutants with Gli1::GFP induced *Ptch1* expression. However, full-length Gli3::GFP was unable to suppress Gli1::GFP-mediated induction of *Ptch1* in the absence of Polaris (*Tg737* ^Δ2–3β-gal^). No expression was seen in controls without reverse transcriptase (−RT). (B) Infection of wild-type cells with a Gli2::GFP expressing adenovirus induced *Ptch1* expression; however, in *Tg737* ^Δ2–3β-gal^ primary limb bud cells, infection with the Gli2::GFP expressing adenovirus failed to induce the pathway, when compared to infection with GFP-only virus (GFP, right lanes). (C) Western blot analysis of proteins isolated from whole E11.5 wild-type embryos (left lane) shows that Gli3 is predominantly found in the processed repressor form (Gli3R). While some Gli3R is evident in the mutant samples, a large proportion of Gli3 remains unprocessed (Gli3A) in *Tg737* ^Δ2–3β-gal^ mutants (right lane). (D) Coinfection of wild-type or *Tg737* ^Δ2–3β-gal^ mutant cells with Gli1::GFP and a truncated Gli3R::GFP indicates that Gli3R is able to repress Gli1-mediated induction of *Ptch1*. Numbers below each lane in (A), (B), and (D) indicate the expression level of *Ptch1* relative to the actin control for the experiment shown.

Genetic studies have indicated that IFT function is required for Shh signaling downstream of *Ptch1,* possibly at the level of Gli function [18–20]. To further explore the connection between Gli activity and Polaris, we used adenoviruses [21] to express the full-length Gli proteins in *Tg737* null cells. Previous results have shown that ectopic expression of Gli1 and Gli2 can induce transcription of Shh target genes while Gli3 has been shown to inhibit Gli1-mediated transcription [8,21,22]. As seen in wild-type cells, infection of *Tg737* ^Δ2–3β-gal^ primary limb cells with full-length Gli1 resulted in increased transcription of *Ptch1* compared to infection with green fluorescent protein (GFP)–only virus (Figure 3A). This indicates that Polaris function is not required for Gli1-mediated pathway activation. However, infection of *Tg737* ^Δ2–3β-gal^ primary limb bud cells with Gli2-expressing virus failed to induce *Ptch1* transcription (Figure 3B) suggesting that Gli2 function requires the activity of Polaris. It is unclear at this time whether the loss of Gli2 function in *Tg737* ^Δ2–3β-gal^ mutants is due to a requirement of Polaris for Gli2 stability or other post-translational regulation. As seen in previous studies, infection of cells with the full-length form of Gli3 was able to repress Gli1-mediated transcription when coexpressed in wild-type cells [22]. However, in cells lacking Polaris, full-length Gli3 failed to repress pathway activation by Gli1, as evidenced by increased *Ptch1* expression (Figure 3A).

### Gli3 Processing Is Inhibited by Loss of Polaris

The above data raised the possibility that loss of Polaris impaired the conversion of the full-length Gli3 to the truncated repressor form. To determine if this was the case, we examined the levels of full-length and processed forms of Gli3 in wild-type and *Tg737* ^Δ2–3β-gal^ whole embryos (E11.5) by Western blot analysis using Gli3 antiserum (gift of B. Wang). In agreement with previously published results [19,20], there was a marked increase in the ratio of the full-length Gli3 to the processed form of Gli3R in *Tg737* mutants (Figure 3C), although some Gli3R is clearly evident. Together, these data suggest that Polaris is required for efficient processing of Gli3.

To determine if the loss of Gli3-mediated repression in *Tg737* ^Δ2–3β-gal^ mutants was due to defects in processing of full-length Gli3 to the repressor form, we infected primary cells with a truncated form of Gli3 (Gli3R) and analyzed the effect on Gli1-mediated transcription. In both wild-type and *Tg737* ^Δ2–3β-gal^ mutant cells, the processed form of Gli3R was able to function as a potent repressor of Gli1-induced transcription of *Ptch1* (Figure 3D). These results indicate that the loss of Gli3 activity observed with the full-length form is due to a defect in processing and not a loss of repressor activity or trafficking to the nucleus.

### Partial Loss of Polaris Function Exacerbates the Phenotype of *Gli3* Heterozygous Mutants

**Figure 4:**
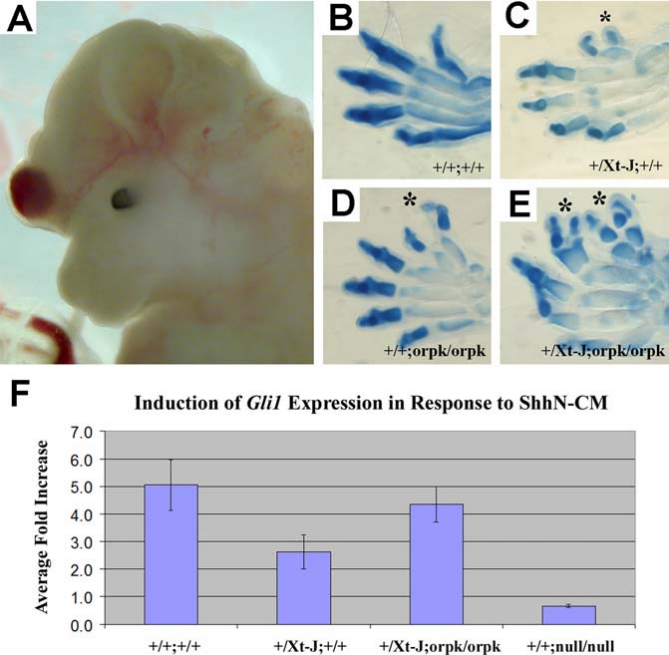
*Gli3^Xt-J/+^*;*Tg737^orpk/orpk^* Embryos Resemble *Gli3^Xt-J/Xt-J^* Null Embryos and Are Responsive to ShhN-CM (A) Example of exencephaly observed in *Gli3^Xt-J/+^*;*Tg737^orpk/orpk^* embryos that is never observed in *Gli3^Xt-J/^* ^+^ or *Tg737^orpk/orpk^* mutants alone. (B–E) Functional interaction of *Gli3* and *Tg737* in digit development. Whereas *Gli3^Xt-J/+^* (C) and *Tg737^orpk/orpk^* (D) embryos each develop one extra preaxial digit (asterisks), *Gli3^Xt-J/+^*;*Tg737^orpk/orpk^* embryos (E) develop multiple ectopic digits compared to wild-type embryos (B). Anterior is to the top. (F) Incubation of cells from *Gli3^+/+^*;*Tg737^+/+^*, *Gli3^Xt-J/+^*;*Tg737^+/+^*, and *Gli3^Xt-J/+^*;*Tg737^orpk/orpk^* mutant mice with ShhN-CM resulted in increased expression of *Gli1* when compared to cells from the same embryo treated with control medium, as determined by quantitative RT-PCR analysis. The results are reported for four littermates of the indicated genotypes. Each sample was analyzed in duplicate, and results are reported as the average fold increase. Unlike *Tg737* ^Δ2–3β-gal^ (null) mutants, which are nonresponsive to ShhN-CM, the *Gli3^Xt-J/+^*;*Tg737^orpk/orpk^* samples are able to respond and activate the pathway, indicating that the *Gli3^Xt-J/+^*;*Tg737^orpk/orpk^* phenotype does not resemble that of *Tg737* ^Δ2–3β-gal^ (null) mutants but rather that of *Gli3^Xt-J/ Xt-J^.*

We predicated that if Polaris is required for proper Gli3 processing, partial loss of Polaris function as seen with the *Tg737^orpk^* hypomorphic allele would exacerbate the phenotype of *Gli3* heterozygous mice and cause a phenotype that is more reminiscent of *Gli3* null mutants. To evaluate this possibility, we crossed *Tg737^orpk/+^* heterozygous mice with *Gli3^Xt-J/+^*;*Tg737^orpk/+^* compound heterozygotes and correlated the resulting phenotypes with the genotypes of the embryos. Heterozygous *Gli3^Xt-J/+^* mice are viable and exhibit a single additional preaxial digit similar to that seen in homozygous *Tg737^orpk/orpk^* mutants [6,12,23]. In contrast, homozygous *Gli3^Xt-J/Xt-J^* mutants are nonviable and have 8–10 nonpatterned digits per limb, as is also seen in *Tg737* ^Δ2–3β-gal^ null mutants [12]. Intriguingly, no viable *Gli3^Xt-J/+^*;*Tg737^orpk/orpk^* offspring were obtained (0/64 pups; seven litters). Analysis at earlier developmental stages indicated that the *Gli3^Xt-J/+^*;*Tg737^orpk/orpk^* mice die during gestation with severe developmental abnormalities including 6–9 digits per limb, exencephaly, abdominal closure defects, and edema (Figure 4A–4E; data not shown). These phenotypes are not characteristic of *Gli3^Xt-J/+^* heterozygous or *Tg737^orpk/orpk^* homozygous mice alone but are seen in *Gli3^Xt-J/Xt-J^* homozygous mutants.

While both *Tg737* ^Δ2–3β-gal^ and *Gli3^Xt-J^* mutant mice have similar digit patterning phenotypes, *Tg737* ^Δ2–3β-gal^ mutants do not express *Ptch1* or *Gli1* in the posterior limb bud while in *Gli3^Xt-J^* mutants the expression domains of *Ptch1* or *Gli1* are expanded. To determine if the limb patterning defects in *Gli3^Xt-J/+^*;*Tg737^orpk/orpk^* mutants were due to a loss of Shh responsiveness, as seen in *Tg737* ^Δ2–3β-gal^ mutants, or more closely resembled the phenotype of *Gli3^Xt-J^* mutants, we tested primary cells derived from these embryos for their ability to induce *Gli1* expression in response to ShhN-CM. In contrast to *Tg737* ^Δ2–3β-gal^ mutants, *Gli3^Xt-J/+^*;*Tg737^orpk/orpk^* cells responded to ShhN-CM with increased expression of *Gli1* when analyzed by quantitative RT-PCR, although at reduced levels compared to wild-type cells (Figure 4F). In agreement with the quantitative RT-PCR data, *Gli1* expression was observed in the posterior region of all embryos by in situ hybridization (data not shown). No overt or consistent differences were evident in *Gli3^Xt-J/+^* or *Gli3^Xt-J/+^*;*Tg737^orpk/orpk^* embryos when compared to wild-type (data not shown). Together these data indicate that the *Gli3^Xt-J/+^*;*Tg737^orpk/orpk^* phenotype more closely resembles that of *Gli3* homozygotes than that of *Tg737* null mutants, which are nonresponsive to ShhN-CM.

### Exogenously Expressed Components of the Shh Signaling Pathway Localize to the Cilia

While it is known that IFT is required for Shh signaling [18,19], it remains unclear whether this is due to a requirement for cilia in Shh pathway activation, production of a secondary signal by cilia, or a novel non-ciliary role for IFT. To begin distinguishing between these possibilities, we evaluated the subcellular localization of several key proteins involved in the Shh signaling pathway, including Gli1, Gli2, Gli3, Gli3R, and Sufu relative to cilia and the IFT protein Polaris.

**Figure 5:**
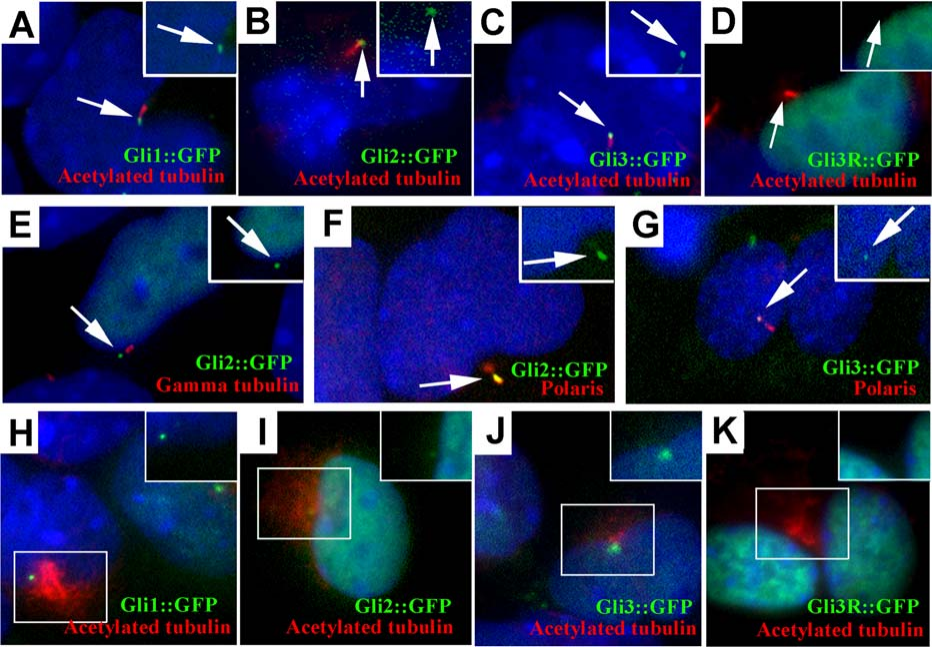
GFP-Tagged Gli Proteins Localize to the Distal Tip of the Cilium in Primary Limb Cell Cultures (A–D) Cells were isolated from limb buds of wild-type embryos at E11.5 and infected with the indicated adenovirus. All three full-length GFP-tagged Gli proteins (green) localize to a domain in the cilium axoneme, which is visualized with anti-acetylated α-tubulin staining (red). In contrast, Gli3R::GFP is restricted to the nucleus and is not detected in this domain (D). (E) The full-length Gli::GFP proteins (Gli2::GFP shown here) do not colocalize with the basal body at the base of the cilium, which is visualized with anti-γ-tubulin staining (red), indicating that the full-length Gli proteins localize to the tips of the cilia. (F and G) Gli2::GFP (F) and Gli3::GFP (G) colocalize with a subdomain of Polaris (red) at the distal tip of the cilium. (H–K) In *Tg737* ^Δ2–3β-gal^ mutant limb bud cells, the GFP-tagged Gli1 (H), Gli2 (I), and Gli3 (J) proteins localize to the nucleus and in the region of stabilized microtubules around the MTOC marked by anti-acetylated α-tubulin. In contrast, the processed form of Gli3 (Gli3R::GFP) (K) is detected only in the nucleus. Insets in all panels show the GFP (green) and nuclear (blue) staining only for the indicated cilium (arrow) or region (box).

In the case of the Gli1, Gli2, Gli3, and Gli3R proteins, localization was determined by infection of primary *Tg737* ^Δ2–3β-gal^ mutant and wild-type limb bud cells with adenoviral vectors that express the full-length Gli proteins or the truncated Gli3R fused to GFP [21]. Infections were performed such that greater than 75% of the cells expressed GFP. For these studies, we focused on cells that had low levels of exogenous expression to minimize any effects that overexpression may have on protein localization. For all three full-length Gli proteins, expression was detected in the nucleus in cells that expressed high levels of GFP (Figure 5; data not shown), as reported previously [22]. However, we also detected a small domain of GFP in all cells expressing the tagged protein that was located near the cilium axoneme as visualized with anti-acetylated α-tubulin antibodies (Figure 5A–5C). The GFP signals failed to colocalize with γ-tubulin (basal body marker), indicating that the Gli::GFP proteins do not localize to the basal body at the base of the cilia (Figure 5E; data not shown). Rather, the GFP signal was found to colocalize with a subdomain of Polaris (Figure 5F and 5G; data not shown)*.* The colocalization of Gli::GFP with a domain of Polaris, but not with γ-tubulin, indicates that the full-length Gli proteins concentrate at the tip of the cilium but not at the base. Treatment of infected cells with ShhN-CM did not alter the distribution of Gli1, Gli2, or Gli3 at the distal tips of cilia (data not shown). However, it may be difficult to assess any changes in localization since GFP is fused to the C-terminus of the Gli proteins. Thus, processing that occurs in the case of full-length Gli3 would remove the GFP tag and prevent visualization of the truncated N-terminal form of the protein that traffics to the nucleus.

In contrast to the localization observed for the three full-length Gli proteins, Gli3R::GFP was detected predominantly in the nucleus. We could detect no GFP signal at the distal tip of cilia, suggesting that after processing, Gli3R is released from the cilia or that the cilia targeting domain is located in the C-terminus of Gli3 (Figure 5D). These possibilities are currently being explored.

In *Tg737* ^Δ2–3β-gal^ mutant cells that lack cilia, the Gli::GFP fusion proteins were seen in the nucleus, as observed in wild-type samples. Additionally, Gli1::GFP, Gli2::GFP, and Gli3::GFP were localized around the MTOC, where the cilia would have formed (Figure 5H–5J). In contrast, Gli3R was present mainly in the nucleus and was not detected around the MTOC in either the wild-type or *Tg737* ^Δ2–3β-gal^ mutant cells (Figure 5D and 5K). The nuclear localization of the Gli::GFP proteins in *Tg737* ^Δ2–3β-gal^ mutants suggests that Polaris is not required for nuclear import of the Gli transcription factors.

### Endogenous Gli3 and Sufu Localize to the Tip of Cilia

**Figure 6:**
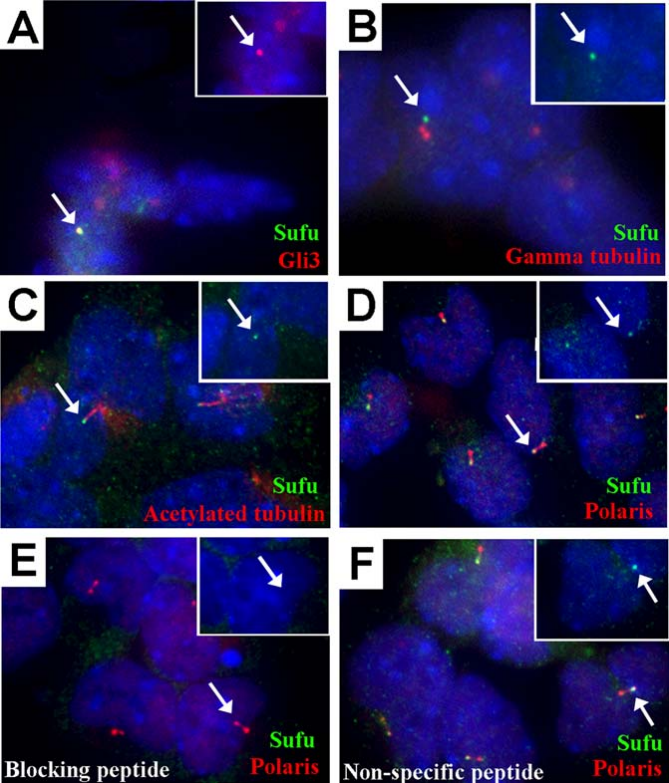
Endogenous Sufu and Gli3 Localize to the Distal Tip of the Cilium in Wild-Type Primary Limb Cell Cultures Sufu (green) and endogenous Gli3 (red) concentrate in the same domain in cultured wild-type limb bud cells (A). As shown for the full-length Gli::GFP proteins, endogenous Sufu does not colocalize with γ-tubulin (red) (B), but is concentrated in a domain at the distal end of the acetylated α-tubulin staining (red) (C). Sufu also partially overlaps with a domain of Polaris (red) (D) in cultured wild-type limb bud cells. Pre-incubation of anti-Sufu antiserum with the immunizing peptide (E), but not with a nonspecific peptide (F), blocks staining at the distal tip of the cilium (anti-Polaris, red; anti-Sufu, green). Inset in all panels shows Gli3 (A) or Sufu (B–F) staining only in the indicated cilium (arrow).

To confirm the localization of GFP-tagged Gli3 at the tip of cilia, and to determine if this was the full-length Gli3 protein or only the GFP-tagged C-terminus, we conducted immunofluorescence analysis of endogenous Gli3 in noninfected primary limb cells using Gli3 antisera generated against the N-terminus of the protein (Figure 6). The data indicate that, as seen with exogenously expressed Gli3::GFP, endogenous Gli3 was concentrated at the tip of cilia (Figure 6A; data not shown). Since the Gli3 antiserum recognizes the N-terminus of Gli3, and GFP is fused to the C-terminus in Gli3::GFP virus, the data suggest that it is the full-length form of Gli3 that localizes to the cilium tip.

Since all three Gli proteins localize to the tip of the cilium and to the nucleus, and since Sufu has been shown to directly interact with the Gli proteins [17], we predicted that Sufu would also be present in these two regions of the cell. To explore this possibility, we analyzed the localization of endogenous Sufu by immunofluorescence in primary limb bud cultures. The data confirm that endogenous Sufu colocalized with endogenous Gli3 and with the Gli::GFP fusion proteins, and partially overlapped with Polaris (Figure 6A and 6D; data not shown). It was also present adjacent to acetylated α-tubulin in the cilium axoneme and did not localize with γ-tubulin at the basal body, again indicating that these proteins concentrate at the tip of the cilium (Figure 6B and 6C). A low level of Sufu was also evident in the nucleus but was in a different plane of focus when the Sufu image for Figure 6 was captured. In addition, Sufu was detected in the cytosol; however, whereas the nuclear and cilium tip signals were blocked by preincubation with the immunizing peptide, the cytoplasmic signal remained unchanged (Figure 6E and 6F), suggesting that it may be nonspecific.

## Discussion

Cilia are expressed on many different cell types in the mammalian body. They are formed and maintained by a highly conserved process termed IFT, but they perform diverse functions on various cell types [1]. In mammals, cilia have been demonstrated to play a critical role in developmental processes—from left–right axis specification and skeletal patterning to normal kidney, pancreas, and liver physiology—as well as disease processes [2–4,6,24]. Recent data have demonstrated that the IFT proteins, which are necessary for cilia formation, are also required for proper limb and neural tube patterning [6,18–20]. Furthermore, the IFT proteins have been shown to function as part of the Shh signal transduction pathway and in regulating Gli activity [19,20]. Here we show that Gli2 and full-length Gli3 function are disrupted in the *Tg737* ^Δ2–3β-gal^ cilium mutants. In contrast, our data indicate that Gli1 and a processed form of Gli3 (Gli3R) are able to induce or repress the Shh pathway, respectively, regardless of the presence or absence of Polaris. Most important, all three full-length Gli::GFP proteins, as well as endogenous Gli3 and Sufu, localize to the distal tip of cilia in primary limb bud cell cultures, supporting a direct role for cilia in regulating Shh signal transduction.

Mice homozygous for the hypomorphic *Tg737^orpk^* allele have preaxial polydactyly, but no alterations in expression of *Shh* or its downstream targets are evident [6]. Preaxial polydactyly is also seen in mice heterozygous for the *Xt-J* allele of *Gli3* [10]. Interestingly, *Gli3^Xt-J/+^*;*Tg737^orpk/orpk^* mice develop multiple ectopic digits on all four limbs and die during gestation. This phenotype is reminiscent of both *Gli3^Xt-J^* and *Tg737* ^Δ2–3β-gal^ homozygous mutants. However, unlike cells from *Tg737* ^Δ2–3β-gal^ mutants, cells from *Gli3^Xt-J/+^*;*Tg737^orpk/orpk^* mice are able to induce *Gli1* transcription in response to ShhN-CM. These data suggest that the hypomorphic *Tg737^orpk^* allele further disrupts Gli3 function and converts the *Gli3^Xt-J/+^* heterozygotes to a phenotype more similar to that seen in the *Gli3* null mutants.

Previous genetic studies on IFT mutant mice have indicated that IFT proteins function in Shh signal transduction at a step downstream from the membrane proteins Ptch and Smoothened and are required for all Gli function [18–20]. In contrast, our data suggest Polaris is required for specific Gli functions since Gli1 and truncated Gli3 (Gli3R) are active in the absence of Polaris while Gli2 and full-length Gli3 are not. These data suggest that the defects observed when mutant cells are infected with full-length Gli3 are likely due to loss of processing of Gli3 to form the repressor, but not loss of repressor activity. While processing of Gli2 remains controversial, Gli2 function is disrupted in *Tg737* ^Δ2–3β-gal^ mice, suggesting that IFT or cilia are required for some aspect of Gli2 regulation or activity. This does not appear to involve translocation of the Gli proteins to the nucleus since they are all detectable in the nucleus of *Tg737* ^Δ2–3β-gal^ mutant cells.

In *Drosophila,* Su(fu) is involved in the negative regulation of Ci (Gli homolog) transcriptional activity by sequestering it in the cytoplasm and targeting it for proteolytic processing to produce a transcriptional repressor [8]. Whether the role of Sufu in targeting Gli proteins for proteolytic processing is conserved in mammals has yet to be determined. Mammalian Sufu has been shown to interact with all three Gli proteins through a conserved SYGH motif in the N-terminus of the Gli proteins in addition to a region in the C-terminus of Gli1, and negatively regulates Gli1 transcriptional activity [16,17]. The colocalization of Sufu and the Gli proteins to the tip of the cilium, along with a requirement for IFT in proper Gli3 processing, suggests that mammalian Sufu may have a similar role in Gli regulation and, furthermore, that proteolytic processing may occur at the tip of the cilium.

Huangfu et al. [18] proposed two possible models for how IFT may regulate Shh signaling, one suggesting the involvement of a cilia-derived signal that is required for Shh pathway activation and a second model in which IFT has two separate functions, one in ciliogenesis and a second one in intracellular transport. While testing these models is hindered by our inability to specifically disrupt cilia formation without also perturbing IFT, the data presented here support a direct role for cilia in Shh pathway regulation. This is based on the localization of multiple components of the Shh pathway in the cilia of wild-type cells, and on altered Gli3 processing and impaired Gli2 function detected in cells lacking this organelle. While we cannot conclusively rule out a non-ciliary function of IFT, we propose that IFT functions to direct and concentrate the Gli proteins, Sufu, and possibly the proteolytic machinery needed for efficient processing of the Gli proteins to a domain located at the distal tips of cilia. In the absence of the cilium, the Gli proteins localize diffusely around the basal body region and fail to undergo normal processing, resulting in their impaired activity.

## Materials and Methods

### Mouse strains and methods.

*Tg737* ^Δ2–3β-gal^, *Tg737^orpk^,* and *Gli3^Xt-J^* mice have been previously described [3,4,23]. *Tg737* ^Δ2–3β-gal^ mice were maintained on a mixed FVB × BALB/c background and genotyped as described [3]. *Tg737^orpk^* mice were maintained on an FVB background and genotyped as described [3]. *Gli3^Xt-J^* mice were maintained on a C57BL/6 background and were genotyped as described [3,25]. Analysis of phenotypes for *Gli3^Xt-J^;Tg737^orpk^* mice was performed on pups or embryos from F_1_ intercrosses. For staged embryos, noon of the day of the vaginal plug appeared was considered E0.5.

In situ hybridization analysis was performed according to standard protocols [26]. *Ptch1* and *Gli1* probes were previously described [27,28].

Skeletal stains were performed as described [29].

### Cell culture.

ShhN-CM and vector-only control conditioned medium were generated as previously described [30]. For induction assays, cells were cultured in a 1:1 mixture of conditioned medium and DMEM + 15% FBS overnight prior to RNA isolation.

Embryos were isolated and identified by phenotype (*Tg737* ^Δ2–3β-gal^ mutants) or by PCR using DNA isolated from yolk sacs. Limb buds for cell culture experiments were removed and treated with 0.25% trypsin in PBS for 15 min at room temperature. Following trypsin treatment, cells were mechanically dissociated and FBS was added to 10%. Cells were collected by centrifugation and plated with DMEM + 15% FBS.

Gli1::GFP, Gli2::GFP, and Gli3::GFP adenovirus constructs encoding C-terminal GFP fusions have been previously described [21]. The Gli3R::GFP adenovirus was generated by replacing the full-length cDNA in the Gli3::GFP vector with the coding region corresponding to amino acids 1–677. This truncated form of Gli3 (Gli3R) has been previously shown to act as a constitutive repressor of Shh signaling [31]. All infections were optimized to produce greater than 75% infection and were done at least three times. To determine if expression levels were consistent between samples, we examined the level of expression of the *Gli::GFP* genes by RT-PCR using primers specific for the GFP coding region. Similar levels of expression were seen in wild-type and mutant samples under the same infection conditions. All localization data shown are representative of the pattern observed in the majority of the cells from all experiments. An identical pattern of localization was observed in the IMCD mouse kidney cell line. No specific localization of GFP was found for cells infected with GFP control virus only.

### RNA isolation and RT-PCR.

RNA was isolated using TRIzol (Invitrogen, Carlsbad, California, United States) according to the manufacturer's instructions. Reverse transcription was performed using SuperScript II reverse transcriptase (Invitrogen) according to the manufacturer's instructions. Equal amounts of cDNA were used as templates for PCR with Taq Polymerase (Brinkman Instruments, Westbury, New York, United States) according to the manufacturer's instructions. Relative expression levels were calculated by comparing the intensity of the *Ptch1* or *Gli1* PCR product to the *actin* PCR product in the same reaction using LabWorks 4.0 software (UVP, Upland, California, United States). Primer sequences are available upon request.

Quantitative RT-PCR measurement was performed using the SmartCycler machine (Cepheid, Sunnyvale, California, United States). The TaqMan primer and probe sets, for *Gli1* and *18S rRNA* (TaqMan Assays-on-Demand Products), were purchased from Applied Biosystems (Foster City, California, United States). The *18S rRNA* gene was used as an internal control. The threshold cycle (*C*_T_) for *Gli1* was first normalized to the corresponding *18S rRNA C*_T_. Relative fold differences were then determined using the 2^(–Δ,Δ[*C*^_T_^])^ method [32] by comparing the expression levels in ShhN-CM-induced cells to their vector conditioned medium controls. No significant difference in basal *Gli1* expression in vector conditioned medium–treated cells was evident between wild-type and *Gli3^Xt-J/+^* samples.

### Immunofluorescence.

For analysis of cilia in vivo, limb buds were dissected from wild-type embryos (E10.5), embedded in OCT, and snap frozen. Sections of 20 μm were cut and stained as previously described [33] using 0.2% Triton X-100 for permeabilization. Cultured primary cells were fixed, permeabilized, and stained using an identical procedure. Anti-Polaris polyclonal antibody was generated by Sigma-Genosys (The Woodlands, Texas, United States) and screened for specificity by Western blot analysis and immunofluorescence. The antiserum recognized a single band of the correct size in wild-type samples by Western blot analysis; this band was absent in *Tg737* ^Δ2–3β-gal^ samples. Only faint nuclear staining was observed in *Tg737* ^Δ2–3β-gal^ primary cells by immunofluorescence, whereas localization to cilia was additionally observed in wild-type samples. Sufu antibody and blocking peptide were obtained from Santa Cruz Biotechnology (Santa Cruz, California, United States). Identical staining with the Sufu antibody was seen in the IMCD mouse kidney cell line. Affinity-purified anti-Gli3 antiserum was provided by B. Wang and used as previously described [9]. Acetylated α-tubulin and γ-tubulin antibodies were obtained from Sigma (St. Louis, Missouri, United States). Gli::GFP fusion proteins were visualized using the GFP tag encoded by the adenovirus constructs. Nuclei were stained with Hoechst 33258 (Sigma).

All fluorescence imaging was performed on a Nikon (Tokyo, Japan) TE200 Eclipse inverted epifluorescence microscope equipped with a CoolSnap HQ cooled CCD camera (Roper Scientific, Trenton, New Jersey, United States) and MetaMorph imaging software (Molecular Devices, Downington, Pennsylvania, United States). All filters and shutters were computer driven.

### Electron microscopy.

Wild-type E11.5 embryos were isolated in PBS and fixed in 2.5% gluteraldehyde in 0.1 M cacodylate buffer for 90 min at room temperature. Samples were washed in three changes of 0.1 M cacodylate buffer, fixed in 1% OsO_4_ for 60 min at room temperature, washed in thee changes of 0.1 M cacodylate buffer, and dehydrated through a graded ethanol series. Samples were processed for scanning or transmission electron microscopy by the University of Alabama at Birmingham High Resolution Imaging Facility using standard procedures. Scanning electron microscopy samples were imaged on an ISI SX-40 scanning electron microscope (Topcon Technologies, Paramus, New Jersey, United States). Transmission electron microscopy sections were imaged on a Zeiss (Oberkochen, Germany) EM 10C transmission electron microscope.

## Abbreviations

E[number]: embryonic day [number]
GFP: green fluorescent protein
Gli: Glioma
IFT: intraflagellar transport
MTOC: microtubule organizing center
Ptch: Patched
RT-PCR: reverse transcription PCR
Shh: Sonic hedgehog
ShhN-CM: ShhN conditioned medium
Sufu: Suppressor of fused
